# Associations of childhood unintentional injuries with maternal emotional status during COVID-19

**DOI:** 10.1186/s12887-021-02846-2

**Published:** 2021-09-24

**Authors:** Xiangrong Guo, Hui Hua, Jian Xu, Zhiwei Liu

**Affiliations:** 1 The International Peace Maternity and Child Health Hospital, Shanghai Jiao Tong University School of Medicine; Shanghai Key Laboratory of Embryo Original Diseases, No. 910 Hengshan Road, Shanghai, 200030 China; 2 MOE-Shanghai Key Lab of Children’s Environmental Health, Xinhua Hospital, Shanghai Jiao Tong University School of Medicine, Shanghai, 200092 China

**Keywords:** COVID-19, Accidents, Child, Maternal emotions, Family characteristics, Public health

## Abstract

**Background:**

To explore the characteristics of unintentional childhood-injury during the COVID-19 pandemic and assess the association of unintentional-injury with maternal emotional status.

**Methods:**

A cross-sectional survey was conducted with a convenience sample of 1300 children under 12-years-old from 21 schools (including nurseries/ kindergartens/ primary schools) in Wuhan and Shanghai during March to April 2020, and the mothers completed questionnaires online. Self-rating Depression/Anxiety Scales were used to evaluate maternal emotional status, questions on child unintentional-injury were based on the International-Statistical-Classification-of-Diseases-and-Related-Health-Problems-version-10 (ICD-10), and a total of 11 kinds of unintentional injuries were inquired. Information on socio-demographic and family-background factors was also collected.

**Results:**

The children of 0–4, 5–9, and 10–12 years accounted for 29.2, 55.2 and 15.6%, respectively, the unintentional-injury rates were 10.29, 4.18 and 3.45%, respectively (*P* < 0.001), and boys had higher rates than girls. The three leading causes included “being struck by/against”, falls and animal bites (traffic-injury accounted for a small proportion). Lower maternal educational, living in suburban/rural (vs. urban) areas, grandparents (vs. mothers) being main caregivers, more child exposure to secondhand smoke, close relatives being suspected/ confirmed COVID-19 cases were associated with a higher risk of child unintentional-injury. After adjusting for related confounders, higher maternal depression levels were associated with a higher risk of unintentional injury.

**Conclusions:**

The characteristics of unintentional childhood injury were different from those in non-pandemic periods. The main causes, risk factors and the association of unintentional injury with maternal depression deserve attention for development of effective measures for preventing children from unintentional injury during COVID-19 pandemic.

**Graphical abstract:**

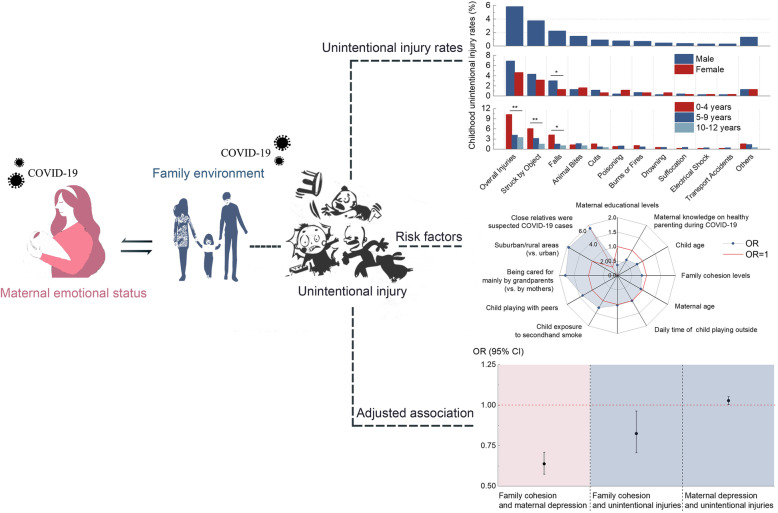

**Supplementary Information:**

The online version contains supplementary material available at 10.1186/s12887-021-02846-2.

## Background

As a public health disaster, the coronavirus disease 2019 (COVID-19) swept across the globe. So far, the situation of the epidemic prevention and control is still not optimistic. Until August 4th, 2021, worldwide COVID-19 cases have exceeded 199 million and deaths were over 4 million [1]. Although several vaccines have been developed and used clinically, the strong transmission ability and rapid mutation speed of the virus make COVID-19 remain a global public health threat [2]. In China, the great pandemic mainly occurred from late January 2020 to the end of April 2020, when school closure and home confinement were both implemented throughout China, and foreign-imported COVID-19 cases have been considered as a main concern since May 2020. From late January 2020 to the end of April 2020 in China, in order to cut off the transmission route of the virus, passenger travel of every type experienced sharp reductions including airports and railway stations being closed and intra-city public transport affected (including in Wuhan and Shanghai) [3], people were requested to stay at home, and school students took online courses at home due to school closure [4, 5].

As defined by the World Health Organization, unintentional injuries occur without any plan or intent [6]. The unintentional injury is the leading cause of death for children, and fatal injuries are actually ‘the tip of the iceberg’ of a range of injuries. According to International Statistical Classification of Diseases and Related Health Problems version-10 (ICD-10)**,** childhood unintentional injuries include falls, being struck by/against, animal bites (including insect stings), transport accidents, drowning, burns or fires, cuts, poisonings, electrical shocks, suffocation and others [7]. More than 10 million children in China suffered from unintentional injuries [8]. Because children were confined at home during the COVID-19 pandemic [9, 10], the characteristics of unintentional childhood injury may be different from those before the pandemic which challenged the prevention of child unintentional-injury in the new situation, however, the new characteristics of unintentional childhood injury (the leading causes, the related social factors such as the associations with age, sex, family types) during the COVID-19 pandemic remain unknown.

Family functioning has significant influencers on the well-beings of children and family members. Although there is no definite definition, it is usually considered that family functioning is about how family members interact, keep relationships, and solve problems. Previous studies reported that the occurrence of unintentional childhood injury was mainly determined by child behavioral attributes and caregiver supervisory patterns [11–13]. During the adversity time, the caregiver-child interaction in the pattern of family functioning may be the main factor influencing child health/ safety. Through questionnaires completed by parents, more family background or social-economic information with potential associations with unintentional childhood injury could be investigated, and moderate injuries (less severe injuries) would not be missed. However, although there have been few studies published on unintentional childhood injury during COVID-19 recently [14, 15], the study data were mainly from public-health/ hospital surveillance systems, and studies based on parental reports were limited.

COVID-19 is a source of unexpected stress and adversity for most of people. Home isolation can prevent the spread of the epidemic, but a large number of studies reported negative psychological effects of isolation [16, 17]. People may feel anxious and/ or depressed, or experience dread, apprehension and fear of impending disaster, as well as a dysphoric mood or loss of interest/ pleasure in usual activities. Studies showed that, during the COVID-19 pandemic, mothers may experience excessive emotional stress [18, 19], probably because of a greater child care burden due to school/ daycare closures and an increased risk of unemployment. Therefore, maternal emotional status during the COVID-19 pandemic deserve special attention [20]. Prior studies suggested that poor maternal emotion regulation led to more problematic behaviors in children [21–23]. However, the association of maternal emotion status with unintentional childhood injury remains unclear.

Therefore, this study aims to explore the characteristics of child unintentional injury during the COVID-19, and the association of maternal emotional status in the pattern of family functioning with unintentional childhood injury, so as to prevent children from unintentional injury during the COVID-19 and develop effective injury prevention strategies.

## Materials and methods

### Study design and participants

This cross-sectional study was conducted online during the COVID-19 pandemic (from March 1th to April 30th, 2020), and involved a convenience sample of 6 schools (1 nurseries/ 4 kindergartens/ 1 primary school) in Wuhan and 15 schools (1 nurseries/ 4 kindergartens/ 10 primary schools) in Shanghai. These schools were regionally and socio-economically diverse in the city and responded positively to the invitation to participate in this study. The online questionnaire was distributed to the mothers of the participating children by the teachers in charge of the classes via WeChat class groups. All the children in these schools were under 12 years old, and all the mothers have electronic devices (cellphones, iPad or computers) to complete the questionnaire. Mothers having any diagnosis of mental illness/ disorder were not invited to participate in this study. Mothers were administered the validated Chinese-version Self-rating Depression/Anxiety Scales (SDS/SAS) and the Family Environment Scale (FES-CV). Mothers were asked to recall whether their children were suffered from unintentional injury since the beginning of the pandemic (in recent 2–3 months). The information on socio-demographic and family background factors was also collected. Before distributing the questionnaire, an introduction about the study and the guidance on how to complete the questionnaire was provided to the mothers of the study children via WeChat class groups. Finally, a total of 1315 questionnaires were collected, and 15 questionnaires were not included because of double submission or logic errors detected during data cleaning. Therefore, 1300 questionnaires were included in the final statistical analysis.

The study was approved by the Research Ethics Committee Board of the International Peace Maternity and Child Health Hospital. The informed consents were obtained from all the study mothers. Patients or the public were not involved in the design, or conduct, or reporting, or dissemination plans of our research.

### Measurement

#### Assessment of maternal emotional status

Maternal depression and anxiety levels were assessed through SDS [24] and SAS [25], respectively. Both scales have good internal consistency (Cronbach’s alphas are both over 0.80). Based on the clinical diagnostic criteria of depression, the SDS is constructed including 20 related items, and the degree of depression is assessed via an index calculated by dividing the SDS total score by 80 and then multiplying by 100. The SAS also includes 20-items. Similarly, the severity of anxiety is assessed through an index calculated via dividing the SAS raw total scores by 80 and then multiplying by 100. SDS and SAS indexes are considered positive for both scores ≥50. Higher SDS and SAS index scores indicate higher depression and anxiety levels.

#### Assessment of family environment

FES-CV is a self-reported measure of family functioning, and is widely used to evaluate the relationship and interaction among family members [26]. The FES-CV composes of ten subscales including cohesion and contradiction subscales, which had acceptable reliability and validity [26]. The cohesion subscale evaluates the extent to which family members describe feelings of closeness, and support or help among family members, while the contradiction subscale evaluates the frequency and intensity of conflict among family members. Higher scores of cohesion and contradiction subscales indicate higher levels of cohesion or contradiction, respectively.

#### Data on unintentional childhood injury

In this study, questions on child unintentional injury since the beginning of the COVID-19 pandemic (since January, 2020) were based on the International Statistical Classification of Diseases and Related Health Problems 10th Revision (ICD-10) [7]. A total of 11 kinds of unintentional injuries were inquired including road traffic injuries, falls, drowning, burns or fires or scald, poisonings, being struck by /against, animal bites (including insect stings), cuts, electrical shocks, suffocation, and others. To be comparable to other studies/ national (or international) reports on unintentional childhood injury, the study children were divided into the following three age groups: 0–4 years, 5–9 years and 10–12 years.

### Covariates

Covariates on child factors included child age, gender, primary caregivers (mother, grandparents or others), daily time the child was exposed to secondhand smoke, daily time child playing with peers, and daily time child playing outside.

Covariates on maternal factors included maternal age, education (primary school, junior high school, senior high school/ technical senior school, college or university, postgraduate level), and knowledge on healthy parenting during COVID-19 pandemic. Other family factors investigated included family structure (three-generation family, nuclear family, separated, divorced, and remarried), home residency (urban, suburban or rural), and whether their close relatives were infected with COVID-19 (no case, suspected case, confirmed case).

### Statistical analysis

The demographic characteristics of the subjects were presented as means and standard deviations (Mean ± SD) or percentages (%). The Chi-square (χ^2^) test was used to compare the rates of child unintentional injuries by age (among three stratified age groups: 0–4 years, 5–9 years and 10–12 years old) and gender. One-way ANOVA (Analysis of variance) were used to compare maternal emotional status/ family cohesion/ contradiction levels between children with and without overall injuries/ main injury causes. The Chi-square (χ^2^) test and multi-variate analyses were both used to analyze the risk factors of childhood unintentional injuries, and multi-variate logistic regression analyses were used to explore the adjusted associations between maternal emotional status, family cohesion/ contradiction and unintentional childhood injuries.

Statistics analyses were performed using SAS 9.4 software (SAS Institute; Cary, NC, USA), and Origin Pro 2020b (Learning Edition, Version 9.7.5.184). Differences were considered significant at *P* < 0.05 (two-tailed).

## Results

### The socio-demographic characteristics of the study children

The socio-demographic characteristics of the study children and their families were shown in Table 1. The percentages of the children in the groups of 0–4 years, 5–9 years, and 10–12 years were 29.2, 55.2 and 15.6%, respectively. The percentages of boys and girls were 53.4 and 46.4%, respectively. A total of 80.0% of the children lived in urban areas. During the COVID-19 pandemic, 86.9% of the study children rarely played with peers. Child daily time on physical activities was 53.1 ± 37.7 min per day.

**Table 1:** The socio- demographic characteristics of the study children (*N* = 1300)

		N (%)		N (%) or (mean ± SD)	
Child Age	≤4 years	379 (29.2%)	Home Residency	Urban	1040 (80.0%)
	5–9 years	718 (55.2%)		Suburban or rural	260 (20.0%)
	10–12 years	203 (15.6%)	Daily Time of Child Playing with Peers	None	917 (70.5%)
Child Gender	Male	694 (53.4%)		Rarely	213 (16.4%)
	Female	606 (46.6%)		General	64 (4.9%)
Family Structure	Nuclear family	768 (59.1%)		Often	61 (4.7%)
	Three generation family	499 (3.4%)		Always	45 (3.5%)
	Others	33 (2.5%)	COVID-19 Infection Among Close Relatives	No	1271 (97.3%)
Maternal Education	Junior high school and below	42 (3.2%)		Suspected cases	11 (0.8%)
	High school or technical school	92 (7.1%)		Confirmed case	25 (1.9%)
	College or university	755 (58.1%)	Maternal Knowledge Levels on Healthy Parenting during COVID-19 ^a^	General	382 (29.5%)
	Postgraduate or above	411 (31.6%)		Very much	915 (70.5%)
Primary Caregivers	Mother	1028 (79.1%)	Daily Time of Child Playing Outside (min/day)		53.05 ± 37.70
	Grandparents	203 (15.6%)	Maternal Age		37.03 ± 5.03
	Others	69 (5.3%)	Maternal Depression		39.25 ± 9.43
Daily Time of Child Exposure to Secondhand Smoke	No	929 (71.5%)	Maternal Anxiety		34.66 ± 7.48
	Rarely	251 (19.3%)	Family Cohesion		8.26 ± 1.27
	Often	120 (9.2%)	Family Contradiction		1.93 ± 1.59

Notes: ^a^ *N* = 1297

A total of 15.7 and 4.1% of the study mothers had positive depression and anxiety scores, while the average SDS and SAS scores were 39.25 ± 9.43 and 34.66 ± 7.48, respectively. Up to 79.1% of mothers and 15.6% of grandparents of the study children were primary caregivers for their children, respectively. The percentages of the mothers with educational levels of junior high school, senior high school (including technical school), college/ university, and postgraduate were 3.2, 7.1, 58.1 and 31.6%, respectively. 29.5% of mothers had little knowledge on how to take care of children during the pandemic.

### The age- and sex-related characteristics of unintentional childhood injury during the COVID-19 pandemic

**Fig. 1:**
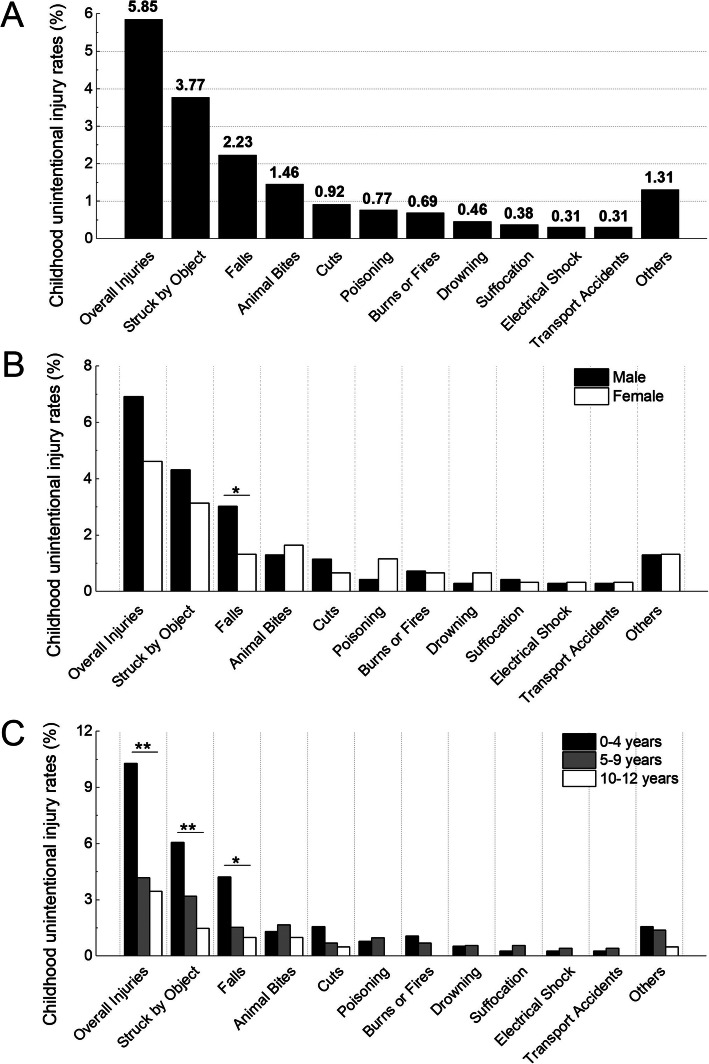
Rates of childhood unintentional injuries during the COVID-19 pandemic. **A**: Rates of childhood unintentional injuries by cause. **B**: Rates of childhood unintentional injuries by sex and cause. **C**: Rates of childhood unintentional injuries by age and cause. Notes: * *P* < 0.05; ** *P* < 0.01

As shown in Fig. 1A, the overall rate of unintentional childhood injury was 5.85%. When calculated by cause, being struck by or against an object had a highest prevalence rate (3.77%), followed by injuries due to falls (2.23%) and animal bites (1.46%). In additional, the prevalence rates of cuts, poisoning, burns or fires, drowning, suffocation, electrical shocks, transport accidents, and other unintentional injuries were 0.92, 0.77, 0.69, 0.46, 0.38, 0.31, 0.31, and 1.31%, respectively.

As illustrated in Fig. 1B, males (6.92%) had a higher rate of the overall unintentional injury than females (4.62%), but the difference didn’t reach statistical significance (*P* = 0.078). For most causes, boys had higher or similar unintentional-injury rates compared with girls. Among the children suffered from the fall injury, the ratio of boy to girl was 72%: 28% (*P* = 0.038). However, for the causes of drowning and poisoning, unintentional-injury rates were a little higher in girls than in boys (*P* > 0.050).

Among the study children, the overall unintentional-injury rates decreased with age. The rate was 10.29% among children aged 0–4 years, 4.18 and 3.45% among children aged 5–9 years and 10–12 years, respectively, and the rate differences reached statistical significance (*P* < 0.001). Among the children less than 4 years old, unintentional injuries were mainly due to being struck by or against an object (6.07%), followed by the causes of falls (4.22%) and cuts (1.58%). In the children aged 5–9 and 10–12 years old, the top cause was still being struck by or against an object (3.20 and 1.48% for children aged 5–9 and 10–12 years, respectively), followed by animal bites (1.67 and 0.99% for children aged 5–9 and 10–12 years, respectively) and falls (1.53 and 0.99% for children aged 5–9 and 10–12 years, respectively). As shown in Fig. 1C, there were statistically significant differences in the rates of being struck by or against an object (*P* = 0.010) and falls (*P* = 0.011) among the three age groups and both rates were highest in the group of 0–4 years. The rate of cuts was highest in the group of 0–4 years (1.58%), followed by the rate in the groups of 5–9 years (0.70%) and 10–12 years (0.49%). Rates of other injuries including animal bites, poisoning, burns or fires, drowning, suffocation, electrical shocks, and transport accidents were not statistically significantly different among the three age groups (*P* > 0.05). Detailed data on the rates of childhood unintentional injuries were presented in Supplemental Material Table 1.

### Risk factors of unintentional childhood injuries during the COVID-19 pandemic

**Fig. 2:**
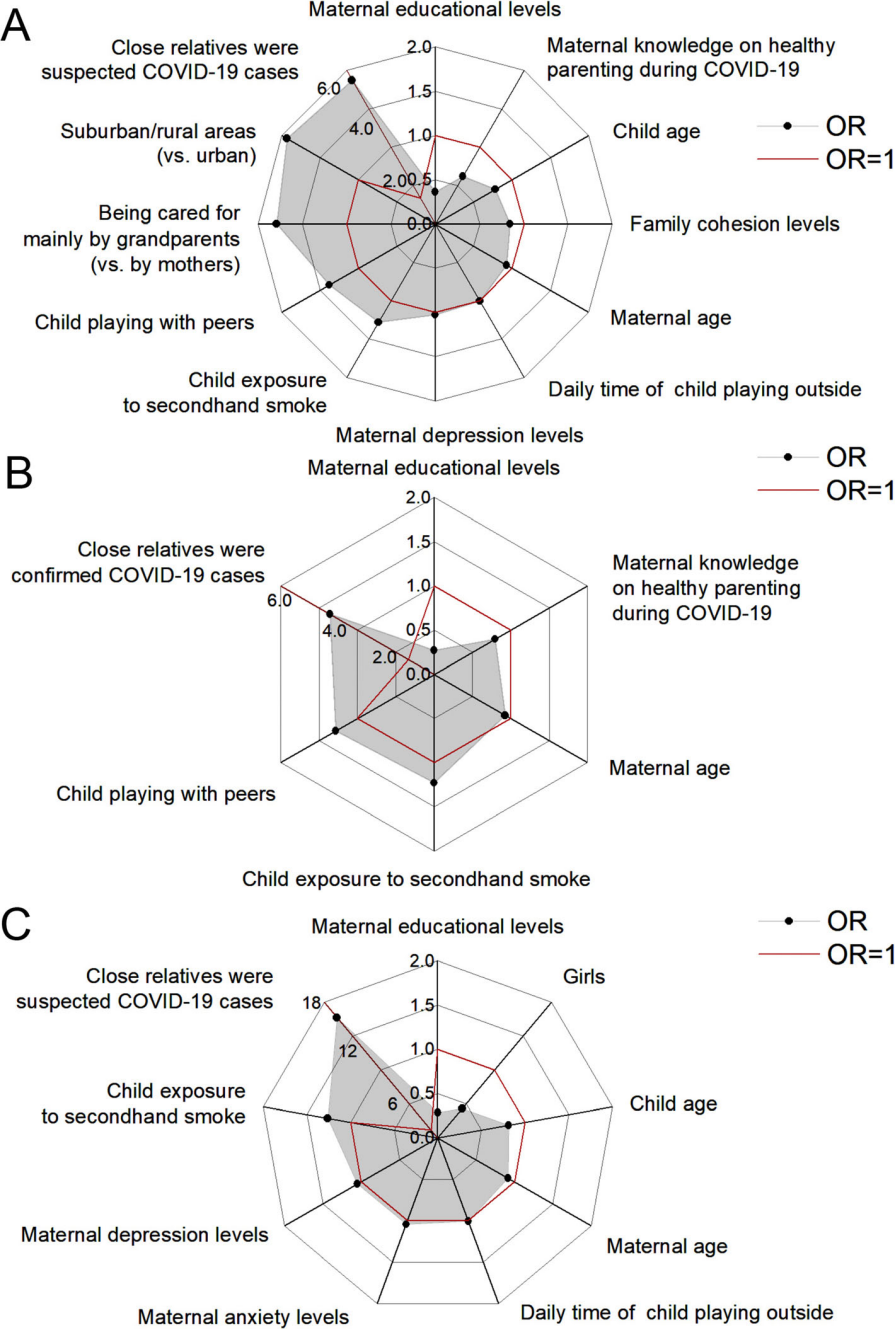
Risk factors of unintentional childhood injuries during the COVID-19 pandemic. **A**: Risk factors of the overall injury. **B**: Risk factors of the injuries due to being struck by or against an object. **C**: Risk factors of the fall injury

As shown in Fig. 2A and Table 2, the risk factors of unintentional injuries included children being cared for mainly by grandparents (vs. by mothers, *P* = 0.041), residency in the suburban/ rural areas (vs. urban areas, *P* = 0.010), lower family cohesion levels (*P* = 0.005), younger children (*P* < 0.001), child having more outdoor time daily (*P* = 0.005), child being exposed to secondhand smoke for more time (*P* < 0.001), more time child playing with peers (*P* < 0.001), close relatives being suspected COVID-19 cases (vs. no cases, *P* = 0.036), younger mothers (*P* = 0.003), lower maternal educational levels (*P* = 0.026) and higher maternal depression levels (*P* = 0.017). Mothers having less knowledge on how to care for children during the pandemic had a marginal association with the occurrence of unintentional injuries (*P* = 0.050).

**Table 2:** The relationships between maternal emotion/ family functioning and unintentional childhood injuries during the COVID-19 pandemic

			Maternal Depression		Maternal Anxiety		Family Cohesion		Family Conflict	
		N (%)	mean ± SD	*P*	mean ± SD	*P*	mean ± SD	*P*	mean ± SD	*P*
Unintentional Injuries	Yes	76 (5.85%)	41.76 ± 10.70	0.017*	35.89 ± 8.50	0.139	7.93 ± 1.43	0.021*	2.09 ± 1.53	0.362
	No	1224 (94.15%)	39.10 ± 9.33		34.59 ± 7.41		8.28 ± 1.26		1.92 ± 1.59	
Being Struck by or Against an Object	Yes	49 (3.77%)	39.51 ± 8.79	0.845	34.63 ± 6.84	0.977	8.22 ± 1.18	0.838	1.88 ± 1.39	0.811
	No	1251 (96.23%)	39.24 ± 9.45		34.66 ± 7.50		8.26 ± 1.27		1.93 ± 1.60	
Falls	Yes	29 (2.23%)	44.24 ± 12.01	0.004**	37.48 ± 9.11	0.040*	7.90 ± 1.59	0.118	2.07 ± 1.62	0.636
	No	1271 (97.77%)	39.14 ± 9.34		34.60 ± 7.43		8.27 ± 1.26		1.93 ± 1.59	

Notes: * *P* < 0.05; ** *P* < 0.01

Analyzed by One-way ANOVA

The risk factors for each cause of unintentional injuries were also analyzed. For child being struck by or against an object, close relatives were confirmed COVID-19 cases (vs. no cases, *P* = 0.027), younger child (*P* < 0.001), child being exposed to secondhand smoke for more time (*P* = 0.029) and more time child playing with peers (*P* = 0.030), younger mothers (*P* = 0.013), lower maternal educational levels (*P* = 0.009) were associated with a higher risk (Fig. 2B and Table 2). For the fall injury, child having more outdoor time daily (*P* = 0.017), younger children (*P* = 0.008), boys (vs. girls, *P* = 0.043), child being exposed to secondhand smoke for more time (*P* = 0.045), close relatives were suspected COVID-19 cases (vs. no cases, *P* = 0.001), younger mothers (*P* = 0.028), lower maternal educational levels (*P* = 0.047), higher levels of maternal depression (*P* = 0.005) and anxiety (*P* = 0.041) were associated with a higher risk (Fig. 2C and Table 2**)**. Detailed data on risk factors for childhood unintentional injuries were presented in Supplemental Material Table 2.

### The relationships between maternal emotional status and unintentional childhood injuries during the COVID-19 pandemic

**Fig. 3:**
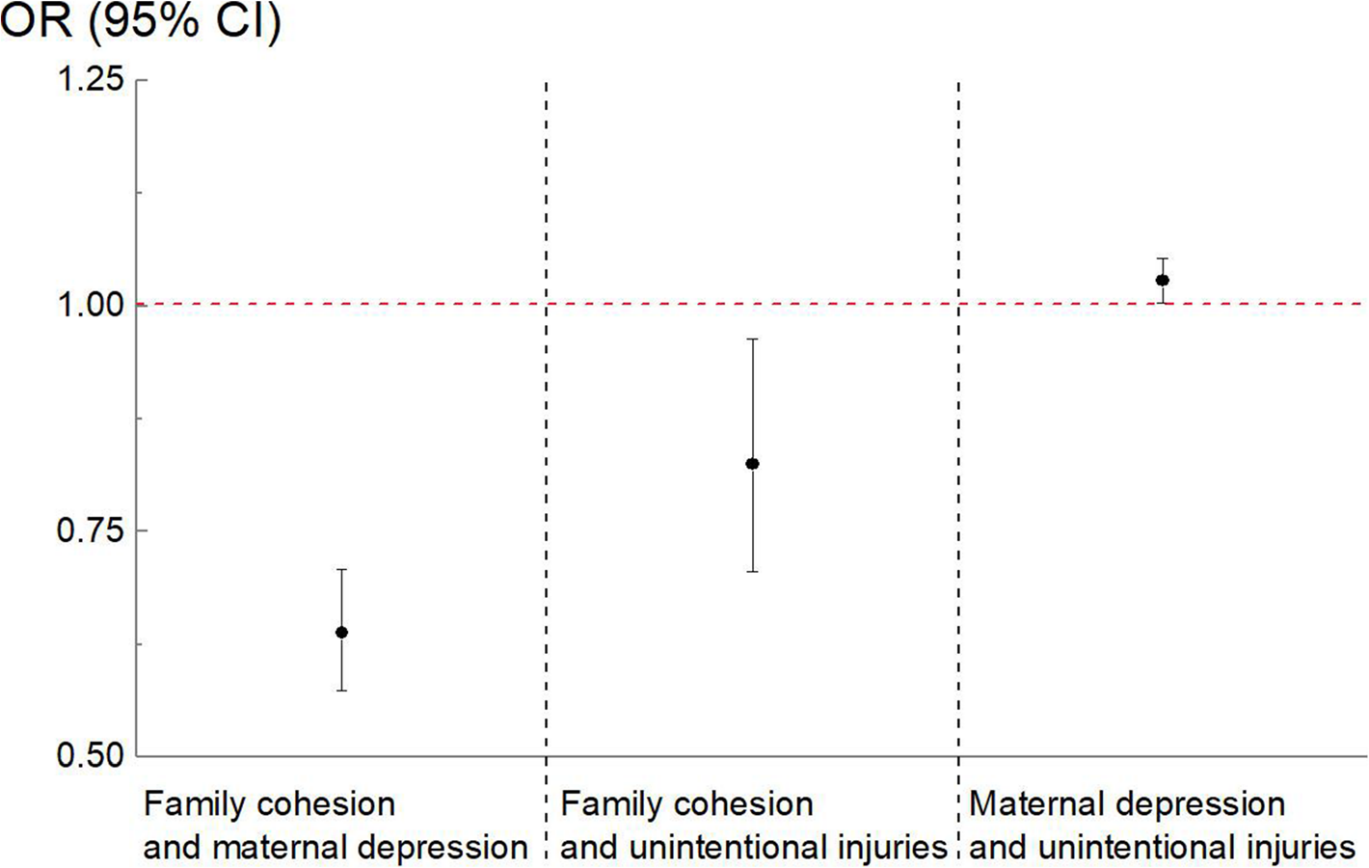
The adjusted relationships among maternal depression levels, family cohesion levels and childhood unintentional injuries during the COVID-19 pandemic

As shown in Table 3, Table 4 and Fig. 3, after adjusting for child age, child gender, maternal age, maternal education, primary caregivers, home residency, and daily time of child playing outside, higher maternal depression levels were associated with a higher risk of the overall unintentional injury (*P* = 0.033). Higher levels of family cohesion were significantly associated with a lower risk of the overall unintentional injury among the study children (*P* = 0.020). However, adjusted models didn’t show significant relationships between maternal emotional status/ family functioning and the unintentional injury due to being struck by or against an object (*P* > 0.05). For the fall injury, higher maternal depression (*P* = 0.008) levels were associated with a higher risk. However, adjusted models didn’t show significant relationships between maternal anxiety and the unintentional injuries (*P* > 0.050). After adjusting for confounders, lower family cohesion levels were significantly associated with higher levels of maternal depression (Table 4 and Supplemental Material Table 3).

**Table 3:** The adjusted relationships between maternal emotion status and childhood unintentional injuries during the COVID-19 pandemic

	Maternal Depression ^a^		Maternal Anxiety ^a^	
	OR (95%CI)	*P*	OR (95%CI)	*P*
Unintentional Injuries	1.027 (1.002, 1.052)	0.033*	1.022 (0.992,1.053)	0.151
Being Struck by or Against an Object	0.997 (0.966,1.029)	0.861	0.998 (0.960,1.039)	0.937
Falls	1.050 (1.013,1.089)	0.008**	1.044 (0.998,1.092)	0.060

Note: ^a^ Adjusted for child age, child gender, maternal age, maternal education, primary caregivers, home residency, and daily time of child playing outside

Analyzed by multi-variate logistic regression analysis. * *P* < 0.05; ** *P* < 0.01

**Table 4:** The adjusted relationships between family functioning and unintentional childhood injuries during the COVID-19 pandemic

	Family Cohesion ^a^		Family Conflict ^a^	
	OR (95%CI)	*P*	OR (95%CI)	*P*
Unintentional Injuries	0.830 (0.709,0.971)	0.020*	1.141 (0.987,1.319)	0.074
Being Struck by or Against an Object	0.977 (0.778,1.226)	0.839	1.040 (0.861,1.255)	0.686
Falls	0.838 (0.656,1.071)	0.158	1.111 (0.881,1.403)	0.374

Note: ^a^ Adjusted for child age, child gender, maternal age, maternal education, primary caregivers, home residency, and daily time of child playing outside

Analyzed by multi-variate logistic regression analysis. * *P* < 0.05

## Discussion

This study is among the first to explore the characteristics of unintentional injury among children aged 0–12 years during the COVID-19 pandemic and the association of maternal emotional status with unintentional injury, which may help to develop a comprehensive child protection strategy that aligns with best practice (including policies and procedures designed to protect children from unintentional injuries during the COVID-19 pandemic).

This study reported that the prevalence rate of maternal depression reached 15.7%. Compared with other studies conducted in China during the COVID-19 pandemic, the depression level in this study was slightly higher than the level reported by Lei (12.93%) [27], and much higher than the levels reported by Peng (6.21%) [28]. Another study proposed that high levels of negative alterations in cognition or mood experienced by women during the COVID-19 pandemic might be due to significant reactivities of neural networks associated with fear processing in females [18]. However, the level of maternal anxiety in this study was similar as the level reported by the previous study during non-epidemic period [29].

This study showed that the ranking of the causes of unintentional childhood injury was different from the ranking before the pandemic. The traffic injury usually remained one of the three leading causes of unintentional childhood injuries in non-pandemic period in most countries. However, in this study, we found that the traffic injury only accounted for 0.31%, less than the rates of poisoning (0.77%), burns or fires (0.69%), drowning (0.46%), suffocation (0.38%), and electrical shock (0.31%). Traffic volumes had experienced a sharp decrease on roads, highways, or airways due to the home quarantine policy during the pandemic [30], perhaps explaining the corresponding decrease in traffic crash. But accordingly, the incidence of accidental injuries at home increased.

Inconsistent with the studies before the pandemic [31], we found that injuries due to “being struck by or against” went up to the top cause of unintentional childhood injury. During the pandemic, children and health-care providers were grounded, which may induce more indoor activities such as pillow fights, hide-and-seek, indoor ball games/ activities, and therefore to induce a higher risk of the “being struck by/ against” injury. Consistent with studies before the pandemic [32, 33], our study found that the fall was still the main cause of unintentional childhood injury (ranked second), especially among children under 4 years old. One global multi-site surveillance study demonstrated that falls, traffic injuries, and burns were the most common unintentional injuries among children ages 0 to 12 years [19]. Another study during 2010–2015 in China demonstrated that, falls accounted for the largest portion of nonfatal injuries, followed by traffic injuries, blunt injuries, and animal bites among children aged 0 to 18 years [31]. Our study was consistent with another retrospective study in Portugal showing that falls increased in the pediatric population during the COVID-19 pandemic [34].

This study found that unintentional injury rates differed by child age, and children aged 0–4 years had the highest rate of unintentional injury, followed by children aged 5–9 years and 10–12 years. Consistent with this study, American and Chinese Centers for Disease Control and Prevention both reported that children aged 0–4 years accounted for the highest proportion of unintentional injury compared with children aged 5–9 years and 10–12 years [31, 35]. In additional, although there were no statistically significant differences between boys and girls in the overall unintentional injury rate during the COVID-19 pandemic, a significant difference was observed for the injury due to falls, and the rate due to falls in boys was nearly 2.5 times higher than that in girls, which may be attributed to the biological, social and cultural differences (for example, boys may be more involved in vigorous physical activities than girls).

This study explored risk factors of unintentional childhood injury during the COVID-19 pandemic. The close relatives being suspected or confirmed COVID-19 cases were found to be associated with a higher risk of injury, which may be because that family members of the COVID-19 cases were quarantined due to close contacts and had psychological distress or panic, and parenting styles were affected, thereby increase the risk of child unintentional injury. This study was consistent with previous studies in the association between more child exposure to secondhand smoke and a higher risk of child behavioral problems. The tobacco smoke exposure was reported to be positively correlated with hyperactivity-impulsivity and conduct problems among children aged 0–4 years [36], hereby might cause an increased risk of unintentional childhood injury [13]. Living in suburban/ rural areas was also associated with a higher risk of unintentional childhood injury, which may be linked to the phenomenon that parents living in suburban/ rural areas may have lower levels of knowledge about injury prevention [37]. More time playing with peers was associated with a higher risk of unintentional injury (especially the injuries related to “being struck by/against”), which may be due to a strong influence of peers on children’s risk-taking behaviors [38]. Grandparents as the main caregivers was also found to be a risk factor of unintentional injury, which may be due to grandparental diminished physical capacity and lower knowledge levels on healthy parenting during the COVID-19 pandemic, therefore to make it difficult to prevent children from unintentional injuries [39]. Consistent with previous studies, we found that older mothers were associated with a lower risk of unintentional childhood injury (especially injuries caused by falls or “being struck by/ against”), which may be because that older mothers may have a greater awareness of the risk of unintentional injury than younger mothers [40]. In line with previous study, this study found that higher levels of maternal knowledge on healthy parenting and higher maternal educational levels were both associated with a lower risk of unintentional injury [41], which may be because that mothers with higher educational or health knowledge levels were more likely to take appropriate behaviors to prevent children from unintentional injury [42].

All the characteristics of unintentional childhood injury during the pandemic remind us of the necessity of focusing on the high-risk children, and strategies are required to improve the risk management. Better education on the prevention of unintentional injury during the pandemic for parents and administrators may bring about improvements in rule modification and facility development.

Consistent with the studies before the pandemic [43–45], this study also reported a significant association of higher levels of maternal depression with a higher risk of unintentional childhood injury. The potential explanations for this association may be as follows: first, maternal negative emotion expression might lead to a worsening inhibitory self-control capacity in children, such as more undisciplined and hyperactive behaviors in children [46]. Previous study demonstrated the associations between higher maternal parenting stress and a higher risk for internalizing problems among preschoolers [47, 48], and children with behavioral problems may be more likely to experience unintentional injuries than normal children. Second, mothers with high depression levels may present as inappropriate supervisory behaviors, less likely to take preventative measures to protect children from injury, and might lead to maternal poor supervision of child risk-taking [43].

Consistent with previous study showing the association between low family cohesion and maternal mental problems [49] and showing negative family functioning and child behavior problems [50], this study found that lower family cohesion was associated with higher maternal depression levels, and also associated with a higher risk of unintentional childhood injury during the COVID-19 pandemic (Supplemental Material Table 3). For typically developing children, high levels of family cohesion are usually associated with high levels of interconnectedness and affiliation among family numbers, and are beneficial to child development [51]. Therefore, we speculate that the possibility of the mediating role of low family cohesion in the association of maternal depression with unintentional childhood injury cannot be excluded, and the family functioning with high levels of family cohesion may break the association between maternal depression and child unintentional injury, and may help prevent children from unintentional injury in pandemic conditions.

The study had limitations. First, the schools were not selected at random, which implied that the mother-child pairs might be not representative of the general population, and selection bias might be induced. Second, the sample size of this study was relatively small, and larger sample size may better describe the characteristics of unintentional childhood injury and define the association of injuries with maternal emotional status. Third, the potential cause-effect relationship between maternal depression and unintentional childhood injury could not be determined due to the cross-sectional design of this study, and maternal depression and unintentional childhood injury may contribute to each other. Fourth, maternal emotional status, family functioning (cohesion/ conflict) and unintentional childhood injury were assessed by the mothers’ reports, and reporter bias may have influenced the results of this study. Previous studies investigating unintentional childhood injury showed that the occurrence of unintentional childhood injury within half-to-1 year could be clearly reported by parents. Although the possibility was relatively low, maternal recall bias on unintentional childhood injury could not be completely excluded [42, 52–54]. Finally, even though we have adjusted for a wide range of confounders, other confounding factors may still exist and may not be included in the study.

## Conclusions

This study reported the characteristics of the unintentional injuries among children aged 0–12 during the COVID-19 pandemic, the potential association between maternal depression and child unintentional injury, and highlighted the importance of the development of a comprehensive planning for preventing children from unintentional injury in exceptional circumstances, such as the quarantine period. Education and prevention on unintentional childhood injury during the pandemic require a comprehensive approach that works at all levels of society—from the mother, family, and community levels to the broader social environment. Our study may help governments or administrators and families develop comprehensive planning for prevention from childhood unintentional injury.

## Supplementary Information

**Additional file 1 : Supplemental Material Table 1**. Number and rates of unintentional injury cases by child gender and age (*N* = 1300). **Supplemental Material Table 2**. Risk factors of childhood unintentional injuries during the COVID-19 pandemic. **Supplemental Material Table 3**. Unadjusted and adjusted relationship between family cohesion and maternal depression/anxiety levels. **Supplemental Material Table 4**. Unadjusted and adjusted relationship between family contradiction and maternal depression/anxiety levels.

## Data Availability

The datasets used and/ or analyzed during the current study are available from the corresponding author on reasonable request.

## Abbreviations

COVID-19: Coronavirus Disease 2019
SDS: Self-rating Depression Scale
SAS: Self-rating Anxiety Scale
FES-CV: Chinese version of Family Environment Scale
ANOVA: Analysis of variance
