# Supplementary material for: Associations of childhood unintentional injuries with maternal emotional status during COVID-19
Source: BMC Pediatr. 2021 Sep 24;21:422. doi: 10.1186/s12887-021-02846-2 (PMC8460849; doi:10.1186/s12887-021-02846-2)
Supplement: Supplementary file 1 — Additional file 1 : Supplemental Material Table 1. Number and rates of unintentional injury cases by child gender and age (N = 1300). Supplemental Material Table 2. Risk factors of childhood unintentional injuries during the COVID-19 pandemic. Supplemental Material Table 3. Unadjusted and adjusted relationship between family cohesion and maternal depression/anxiety levels. Supplemental Material Table 4. Unadjusted and adjusted relationship between family contradiction and maternal depression/anxiety levels. [file 12887_2021_2846_MOESM1_ESM.docx]

**Supplemental Material**

**Supplemental Material Table 1: Number and rates of unintentional injury cases by child gender and age (N=1300).**

|  | All |  | Gender | | |  | Age | | | |
| --- | --- | --- | --- | --- | --- | --- | --- | --- | --- | --- |
|  |  |  | Male | Female | *P* |  | 0-4 years | 5-9 years | 10-12 years | *P* |
|  | n=1300 |  | n=694 | n=606 |  |  | n=379 | n=718 | n=203 |  |
| Unintentional Injuries | 76(5.85%) |  | 48(6.92%) | 28(4.62%) | 0.078 |  | 39(10.29%) | 30(4.18%) | 7(3.45%) | <0.001** |
| Struck by or Against an Object | 49(3.77%) |  | 30(4.32%) | 19(3.14%) | 0.262 |  | 23(6.07%) | 23(3.20%) | 3(1.48%) | 0.010** |
| Falls | 29(2.23%) |  | 21(3.03%) | 8(1.32%) | 0.038* |  | 16(4.22%) | 11(1.53%) | 2(0.99%) | 0.011* |
| Animal Bites | 19(1.46%) |  | 9(1.30%) | 10(1.65%) | 0.596 |  | 5(1.32%) | 12(1.67%) | 2(0.99%) | 0.731 |
| Cuts | 12(0.92%) |  | 8(1.15%) | 4(0.66%) | 0.354 |  | 6(1.58%) | 5(0.70%) | 1(0.49%) | 0.298 |
| Poisoning | 10(0.77%) |  | 3(0.43%) | 7(1.16%) | 0.242 |  | 3(0.79%) | 7(0.97%) | 0(0.00%) | 0.173 |
| Burns or Fires | 9(0.69%) |  | 5(0.72%) | 4(0.66%) | 1.000 |  | 4(1.06%) | 5(0.70%) | 0(0.00%) | 0.179 |
| Drowning | 6(0.46%) |  | 2(0.29%) | 4(0.66%) | 0.564 |  | 2(0.53%) | 4(0.56%) | 0(0.00%) | 0.359 |
| Suffocation | 5(0.38%) |  | 3(0.43%) | 2(0.33%) | 1.000 |  | 1(0.26%) | 4(0.56%) | 0(0.00%) | 0.330 |
| Electrical Shock | 4(0.31%) |  | 2(0.29%) | 2(0.33%) | 1.000 |  | 1(0.26%) | 3(0.42%) | 0(0.00%) | 0.465 |
| Transport Accidents | 4(0.31%) |  | 2(0.29%) | 2(0.33%) | 1.000 |  | 1(0.26%) | 3(0.42%) | 0(0.00%) | 0.465 |
| Others | 17(1.31%) |  | 9(1.30%) | 8(1.32%) | 0.971 |  | 6(1.58%) | 10(1.39%) | 1(0.49%) | 0.445 |

Notes: * *P* <0.05, ** *P* <0.01

Analyzed by Chi-square test.

**Supplemental Material Table 2: Risk factors of childhood unintentional injuries during the COVID-19 pandemic.**

| Variables | Unintentional Injuries | |  | Being Struck by or Against an Object | |  | Falls | |
| --- | --- | --- | --- | --- | --- | --- | --- | --- |
|  | OR (95%CI) | *P* |  | OR (95%CI) | *P* |  | OR (95%CI) | *P* |
| Child Age (years) | 0.782 (0.708, 0.864) | <0.001** |  | 0.794 (0.704, 0.897) | <0.001** |  | 0.812 (0.696, 0.947) | 0.008** |
| Child Gender |  |  |  |  |  |  |  |  |
| Male | REF | REF |  | REF | REF |  | REF | REF |
| Female | 0.652 (0.404, 1.053) | 0.080 |  | 0.716 (0.399, 1.286) | 0.264 |  | 0.429 (0.189, 0.975) | 0.043* |
| Maternal Age (years) | 0.929 (0.885, 0.975) | 0.003** |  | 0.927 (0.873, 0.984) | 0.013* |  | 0.916 (0.848, 0.991) | 0.028* |
| Maternal Education |  |  |  |  |  |  |  |  |
| Junior high school and below | REF | REF |  | REF | REF |  | REF | REF |
| High school or technical school | 0.273 (0.073, 1.024) | 0.054 |  | 0.249 (0.057, 1.098) | 0.066 |  | 0.143 (0.014, 1.416) | 0.096 |
| College/university or above | 0.360 (0.146, 0.885) | 0.026* |  | 0.270 (0.101, 0.722) | 0.009** |  | 0.285 (0.082, 0.983) | 0.047* |
| Primary Caregivers |  |  |  |  |  |  |  |  |
| Mother | REF | REF |  | REF | REF |  | REF | REF |
| Grandparents | 1.790 (1.025, 3.125) | 0.041* |  | 1.069 (0.491, 2.326) | 0.867 |  | 1.800 (0.751, 4.315) | 0.188 |
| Others | 1.437 (0.555, 3.721) | 0.454 |  | 1.184 (0.356, 3.938) | 0.783 |  | 1.504 (0.344, 6.572) | 0.587 |
| Home Residency |  |  |  |  |  |  |  |  |
| Urban | REF | REF |  | REF | REF |  | REF | REF |
| Suburban or rural | 1.932 (1.167, 3.199) | 0.010** |  | 1.812 (0.971, 3.379) | 0.062 |  | 1.540 (0.674, 3.518) | 0.305 |
| COVID-19 Infection Among Close Relatives | |  |  |  |  |  |  |  |
| No | REF | REF |  | REF | REF |  | REF | REF |
| Suspected but excluded | 5.628 (1.116, 28.379) | 0.036* |  | 3.886 (0.468, 32.253) | 0.209 |  | 15.936 (3.070, 82.709) | 0.001** |
| Confirmed case | 2.532 (0.735, 8.720) | 0.141 |  | 4.080 (1.170, 14.233) | 0.027* |  | 2.173 (0.282, 16.734) | 0.456 |
| Maternal Knowledge Levels on Healthy Parenting during COVID-19 | 0.621 (0.386, 1.000) | 0.050* |  | 0.778 (0.427, 1.419) | 0.413 |  | 0.677 (0.317, 1.447) | 0.314 |
| Daily Time of Child Playing Outside(min/day) | 1.007 (1.002, 1.011) | 0.005** |  | 1.004 (0.998, 1.010) | 0.191 |  | 1.008 (1.001, 1.014) | 0.017* |
| Daily Time of Child exposure to Secondhand Smoke (h/day) | 1.283 (1.107, 1.487) | <0.001** |  | 1.230 (1.021, 1.483) | 0.029* |  | 1.262 (1.005, 1.584) | 0.045* |
| Daily Time of Child Playing with Peers | 1.381 (1.157, 1.649) | <0.001** |  | 1.283 (1.024, 1.607) | 0.030* |  | 1.106 (0.798, 1.534) | 0.545 |
| Family Cohesion | 0.843 (0.728, 0.976) | 0.023* |  | 0.978 (0.786, 1.216) | 0.838 |  | 0.838 (0.670, 1.048) | 0.122 |
| Family Contradiction | 1.067 (0.929, 1.225) | 0.362 |  | 0.978 (0.814, 1.174) | 0.811 |  | 1.055 (0.846, 1.316) | 0.636 |
| Maternal Depression | 1.028 (1.005, 1.052) | 0.017* |  | 1.003 (0.973, 1.034) | 0.845 |  | 1.051 (1.015, 1.087) | 0.005** |
| Maternal Anxiety | 1.022 (0.993, 1.052) | 0.140 |  | 0.999 (0.962, 1.038) | 0.977 |  | 1.045 (1.002, 1.090) | 0.041* |
|  |  |  |  |  |  |  |  |  |

Notes: * *P* <0.05; ** *P* <0.01.

Analyzed by uni-variate logistic regression analysis.

**Supplemental Material Table 3: Unadjusted and adjusted relationship between family cohesion and maternal depression/anxiety levels**.

|  | Family Cohesion | | Family Cohesion ^a^ | |
| --- | --- | --- | --- | --- |
|  | OR (95%CI) | *P* | OR (95%CI) | *P* |
| Maternal Depression | 0.634 (0.573, 0.702) | <0.001** | 0.638 (0.574, 0.708) | <0.001** |
| Maternal Anxiety | 0.659 (0.574, 0.756) | <0.001** | 0.655 (0.568, 0.755) | <0.001** |

Notes: ^a^ Adjusted for child gender, maternal age, maternal education, home residency, and maternal knowledge levels on healthy parenting during COVID-19. ** *P* <0.01.

Analyzed by uni-variate and multi-variate logistic regression analyses.

**Supplemental Material Table 4: Unadjusted and adjusted relationship between family contradiction and maternal depression/anxiety levels**.

|  | Family Contradiction | | Family Contradiction ^a^ | |
| --- | --- | --- | --- | --- |
|  | OR (95%CI) | *P* | OR (95%CI) | *P* |
| Maternal Depression | 1.286 (1.181, 1.402) | <0.001** | 1.321(1.209, 1.445) | <0.001** |
| Maternal Anxiety | 1.522 (1.327, 1.745) | <0.001** | 1.533(1.331, 1.765) | <0.001** |

Notes: ^a^ Adjusted for child gender, maternal age, maternal education, home residency, and maternal knowledge levels on healthy parenting during COVID-19. ** *P* <0.01.

Analyzed by uni-variate and multi-variate logistic regression analyses.
